# Supplementary material for: Mechanisms governing the pioneering and redistribution capabilities of the non-classical pioneer PU.1
Source: Nat Commun. 2020 Jan 21;11:402. doi: 10.1038/s41467-019-13960-2 (PMC6972792; doi:10.1038/s41467-019-13960-2)
Supplement: Supplementary file 7 — Source data [file 41467_2019_13960_MOESM7_ESM.zip › Source_Data/FigureS2/FigureS2B_MotifScanOutput/KmeansCluster13/homerResults/motif19.similar.html]

motif19

## Information for motif19

A
T
G
C
G
A
C
T
C
A
G
T
T
C
G
A
C
A
G
T
G
T
A
C
C
G
T
A
T
A
C
G
G
T
C
A
C
G
T
A
  
Reverse Opposite:  

G
C
A
T
C
A
G
T
A
T
G
C
G
C
A
T
C
A
T
G
G
T
C
A
A
G
C
T
G
T
C
A
C
T
G
A
T
A
C
G
  

|  |  |
| --- | --- |
| p-value: | 1e-25 |
| log p-value: | -5.813e+01 |
| Information Content per bp: | 1.521 |
| Number of Target Sequences with motif | 160.0 |
| Percentage of Target Sequences with motif | 9.46% |
| Number of Background Sequences with motif | 1629.5 |
| Percentage of Background Sequences with motif | 3.61% |
| Average Position of motif in Targets | 99.6 +/- 58.1bp |
| Average Position of motif in Background | 100.7 +/- 63.7bp |
| Strand Bias (log2 ratio + to - strand density) | -0.1 |
| Multiplicity (# of sites on avg that occur together) | 1.02 |
| Motif File: | file (matrix) reverse opposite |

### Similar de novo motifs found

|  |  |  |  |  |  |  |  |
| --- | --- | --- | --- | --- | --- | --- | --- |
| Rank | Match Score | Redundant Motif | P-value | log P-value | % of Targets | % of Background | Motif file |
| 1 | 0.785 | G C A T C A T G C T G A A G C T G A C T T C G A A T C G | 1e-18 | -43.580646 | 13.77% | 7.28% | motif file (matrix) |
| 2 | 0.834 | A G T C A C T G T A G C G A C T C G A T G C T A C G A T A G T C G C T A T A C G A G T C | 1e-15 | -35.044317 | 3.19% | 0.80% | motif file (matrix) |
